# A Context-Informed Framework for Interpreting Iron Status in Heterogeneous Settings: Insights from Peru

**DOI:** 10.3390/nu18193152

**Published:** 2026-09-24

**Authors:** Jeannett Rivera-Manhualaya, Parminder S. Suchdev, Chris A. Rees, Kenia Lobo, Yi-An Ko, Melissa F. Young, Gustavo F. Gonzales, Cinthya Vásquez-Velasquez

**Affiliations:** 1 Facultad de Ciencias e Ingeniería, Universidad Peruana Cayetano Heredia, Lima 15102, Peru; jeannett.rivera.m@upch.pe; 2 Hubert Department of Global Health, Rollins School of Public Health, Emory University, Atlanta, GA 30322, USA; parmi.suchdev@emory.edu (P.S.S.); melissa.young@emory.edu (M.F.Y.); 3 Department of Pediatrics, Emory University, Atlanta, GA 30322, USA; 4 Division of Pediatric Emergency Medicine, School of Medicine, Emory University, Atlanta, GA 30322, USA; chris.rees@emory.edu (C.A.R.); kloboar@emory.edu (K.L.); 5 Department of Emergency Medicine, Children’s Healthcare of Atlanta, Atlanta, GA 30342, USA; 6 Department of Biostatistics and Bioinformatics, Rollins School of Public Health, Emory University, Atlanta, GA 30322, USA; yi-an.ko@emory.edu; 7 Academia Nacional de Medicina, Lima 15074, Peru; gonzalesrengifogustavo@gmail.com; 8 Carrera de Ingeniería Biomédica, Facultad de Ingeniería e Inteligencia Artificial, Universidad San Ignacio de Loyola, Lima 15024, Peru

**Keywords:** iron deficiency, inflammation, anemia, iron metabolism, BRINDA, Peru

## Abstract

Iron deficiency (ID), with or without anemia, remains a major global public health challenge, particularly in populations exposed to inflammatory conditions. Accurate assessment of iron status is difficult because ferritin, the primary biomarker of iron stores, is also an acute-phase reactant that increases during inflammation, potentially masking underlying ID and leading to diagnostic misclassification. The Biomarkers Reflecting Inflammation and Nutritional Determinants of Anemia (BRINDA) approach uses regression-based methods incorporating *C*-reactive protein (CRP) and α1-acid glycoprotein (AGP) to adjust iron biomarkers for inflammation. However, its performance in settings characterized by chronic low-grade inflammation, obesity, altitude-related physiology, and environmental stressors remains insufficiently characterized. We propose a context-informed conceptual framework that complements established BRINDA adjustment by considering chronic low-grade inflammation, candidate composite inflammatory markers, and contextual modifiers that may influence the interpretation of iron status. This framework incorporates established and candidate biomarkers, including CRP, AGP, the systemic immune-inflammation index (SII), and the neutrophil-to-lymphocyte ratio (NLR), together with contextual factors such as altitude, metabolic status, and environmental exposures. We also discuss the complex interplay between inflammation and iron metabolism through hepcidin dysregulation and oxidative stress, which may complicate the interpretation of ferritin and other iron biomarkers. Using Peru as an illustrative setting of heterogeneous epidemiological and environmental conditions, we describe how coastal, high-altitude, and Amazonian populations may be differentially affected by these interactions. This context-informed framework provides a structured basis for investigating whether chronic inflammatory and contextual factors add interpretative value beyond established approaches. Prospective and external validation across diverse populations will be required before clinical or public health implementation.

## 1. Introduction

Iron deficiency (ID), with or without anemia, remains a major global public health challenge, particularly in populations where inflammation is prevalent [1]. Accurate assessment of iron status in these settings is inherently complex, as serum ferritin, the primary biomarker of iron stores, is also an acute-phase reactant that increases in response to inflammatory processes. As a result, ferritin may overestimate iron stores and mask underlying deficiency, leading to systematic misclassification with important clinical and public health implications [2,3]. Despite decades of iron supplementation programs, childhood anemia remains highly prevalent in many low- and middle-income countries, suggesting that mechanisms beyond iron deficiency contribute substantially to its persistence.

To address this limitation, the Biomarkers Reflecting Inflammation and Nutritional Determinants of Anemia (BRINDA) regression correction approach was developed to adjust iron biomarkers for the effects of inflammation using *C*-reactive protein (CRP) and α1-acid glycoprotein (AGP) [2,4]. While this statistical adjustment has significantly improved the estimation of iron deficiency (ID) in settings where infectious diseases are common, its performance in populations where inflammatory burden is predominantly chronic, low-grade, metabolic, or modified by environmental and physiological stressors is less well established.

In many populations, nutritional and epidemiological transitions, obesity, metabolic disorders, subclinical infections, environmental exposures, and physiological conditions such as high-altitude hypoxia frequently coexist and may contribute to chronic low-grade inflammation. Unlike the acute inflammatory responses typically associated with infectious diseases, this inflammation is often systemic and subclinical, creating a more complex physiological context in which the interpretation of iron biomarkers becomes particularly challenging.

Within this context, this article critically examines the interaction between chronic low-grade inflammation and iron metabolism and proposes a context-informed interpretative framework that complements established BRINDA adjustment in heterogeneous epidemiological and physiological settings. Building on BRINDA’s regression-based use of continuous inflammatory biomarkers, the framework proposes established and candidate inflammatory markers, iron biomarkers, and relevant contextual modifiers within a structured interpretative approach. It does not modify the BRINDA regression-correction methodology or constitute a new statistical correction equation or validated diagnostic model. Rather, it provides a hypothesis-generating framework for investigating whether additional inflammatory and contextual factors add interpretative value beyond established approaches, an objective that requires prospective validation.

## 2. Literature Search and Evidence Synthesis

This structured narrative review was developed through a literature search focused on the interaction between inflammation and iron metabolism, the interpretation of iron biomarkers in inflammatory states, the BRINDA methodology, composite inflammatory indices, and contextual modifiers relevant to heterogeneous populations. PubMed/MEDLINE and Scopus were searched, with the final search updated on 16 September 2026.

The search strategy was organized around four predefined conceptual domains: (1) biological mechanisms linking inflammation and iron homeostasis; (2) epidemiological associations between inflammatory and iron biomarkers; (3) methods for inflammation adjustment and iron-status classification; and (4) contextual factors potentially modifying these relationships. Search concepts included combinations of terms related to iron deficiency, anemia, ferritin, soluble transferrin receptor, total body iron, transferrin saturation, hepcidin, inflammation, *C*-reactive protein, α1-acid glycoprotein, BRINDA, systemic immune-inflammation index, neutrophil-to-lymphocyte ratio, systemic inflammation response index, obesity, metabolic inflammation, altitude, hypoxia, infection, environmental exposures, and Peru. The complete database-specific search strategies are provided in Supplementary Table S1.

Eligible publications included systematic and narrative reviews, international guidelines, population-based observational studies, and mechanistic or longitudinal studies relevant to at least one of the four predefined domains. Publications in English or Spanish were considered. World Health Organization guidelines and national epidemiological reports from Peru were additionally reviewed as authoritative sources. Reference lists of key reviews, methodological papers, and relevant primary studies were also examined to identify additional publications pertinent to the predefined domains.

Titles and abstracts were initially screened for relevance to the objectives of the review. Full texts of potentially eligible publications were subsequently assessed for their contribution to the predefined conceptual domains. Priority was given to studies providing direct biological, epidemiological, methodological, or contextual evidence relevant to the relationships represented in the proposed framework.

Because this was a structured narrative review rather than a systematic review, study selection was guided by relevance to the predefined conceptual domains rather than by a formal systematic-review protocol. No meta-analysis or quantitative evidence synthesis was performed. Given the heterogeneity of study designs, populations, biomarkers, exposures, and outcomes, the evidence was synthesized narratively. Accordingly, the proposed context-informed framework should be regarded as hypothesis-generating rather than as a validated diagnostic model.

The methodological quality and reporting of the final revised manuscript were independently assessed by two authors using the Scale for the Assessment of Narrative Review Articles (SANRA) [5]. Details of its application, item-level scores and justifications, and the overall SANRA score (12/12) are provided in Supplementary Table S2.

## 3. Chronic Low-Grade Inflammation

Chronic low-grade inflammation is a persistent, systemic, and subclinical condition characterized by sustained alterations in inflammatory mediators, including interleukin-6 (IL-6) and tumor necrosis factor-alpha (TNF-α), and modest elevations in inflammatory markers such as CRP or AGP and, in some settings, erythrocyte sedimentation rate (ESR) [6,7,8,9]. This state of chronic low-grade inflammation is closely linked to non-communicable diseases and represents a common pathophysiological phenomenon observed in conditions including obesity, type 2 diabetes, hypertension, and atherosclerosis [10,11,12].

Unlike acute inflammation, which is typically self-limited and homeostatic, chronic low-grade inflammation reflects a sustained disruption of metabolic and immune balance. It is associated with modern lifestyle factors, including excess caloric intake, physical inactivity, chronic stress, and sleep disturbances, which interact with underlying genetic susceptibility [13,14]. Mechanistically, processes such as adipose tissue hypoxia, macrophage infiltration, and metabolic stress contribute to chronic low-grade inflammation [15].

A key consequence of this inflammatory state is the dysregulation of iron homeostasis. Inflammatory signaling induces hepcidin production, which reduces intestinal iron absorption and promotes iron sequestration in macrophages and hepatocytes, thereby limiting systemic iron availability [16]. This process may contribute to functional ID and inflammation-related anemia. Chronic low-grade inflammation also alters iron metabolism and increases inflammatory markers, complicating the differentiation between absolute iron deficiency and inflammation-mediated iron sequestration. For example, in young individuals with obesity, reduced serum iron levels have been reported alongside increased inflammatory markers and a higher risk of anemia [17].

Importantly, iron biomarkers must be interpreted in the biological context of chronic low-grade inflammation. The persistent interaction between inflammatory pathways and iron metabolism creates a complex physiological environment that challenges conventional diagnostic approaches and underscores the need for integrated interpretative frameworks.

## 4. Iron Biomarkers

Assessment of iron status relies on multiple biomarkers that reflect distinct compartments of iron metabolism. Among these, serum ferritin is the most widely used indicator due to its ability to estimate body iron stores [18]; however, its interpretation can be significantly limited in inflammatory conditions.

### 4.1. Ferritin

Ferritin is an intracellular iron storage protein composed of 24 subunits, including heavy (H) and light (L) chains encoded by the FTH1 and FTL genes [19]. H-ferritin exhibits ferroxidase activity, facilitating the conversion of ferrous to ferric iron and limiting oxidative damage, whereas L-ferritin promotes stable iron storage.

Under inflammatory conditions, ferritin expression is modulated by pro-inflammatory cytokines, including IL-1β, IL-6, and TNF-α, through transcriptional and post-transcriptional mechanisms involving inflammatory signaling pathways such as NF-κB [20].

In this context, ferritin extends beyond its storage function and acts as an inflammatory mediator, contributing to immune response amplification [21]. Moreover, the H- and L-ferritin subunits have distinct but complementary roles in iron homeostasis. H-ferritin possesses ferroxidase activity, facilitating the oxidation of Fe^2+^ to Fe^3+^ and limiting iron-mediated oxidative damage, whereas L-ferritin promotes iron nucleation and stable mineralization within the ferritin core, contributing to long-term iron storage [22,23].

### 4.2. Hepcidin

Hepcidin is currently recognized as the master regulator of systemic iron homeostasis [24]. It inhibits intestinal iron absorption and macrophage iron release through degradation of ferroportin. Hepcidin concentrations increase during inflammation, particularly in response to IL-6 signaling, contributing to functional iron deficiency despite adequate or increased iron stores. Although hepcidin has potential utility for distinguishing iron deficiency from inflammation-mediated iron sequestration, its clinical application remains limited because assays lack international standardization and are not routinely available in most healthcare settings [25].

### 4.3. Other Iron Biomarkers

The soluble transferrin receptor (sTfR) reflects cellular iron demand and was initially considered less influenced by inflammation; however, emerging evidence indicates that sTfR concentrations may also be altered in inflammatory states [26,27]. Transferrin saturation, derived from the ratio of serum iron to transferrin, provides an estimate of circulating bioavailable iron [28].

Total body iron (TBI), estimated from the ratio of soluble transferrin receptor to serum ferritin, provides a quantitative assessment of iron status across a continuum from iron deficiency to iron repletion [29,30]. Nevertheless, its interpretation may also be compromised in chronic low-grade inflammation because both ferritin and sTfR can be influenced by inflammatory processes [27].

Overall, chronic low-grade inflammation systematically alters the interpretation of iron biomarkers by affecting both their regulation and physiological significance. In this context, biomarkers such as ferritin no longer exclusively reflect iron stores but instead integrate complex metabolic and inflammatory signals, complicating the distinction between iron deficiency and inflammation-related anemia [31,32]. Each biomarker provides complementary information regarding iron metabolism but also presents specific limitations that become particularly relevant under inflammatory conditions (Table 1). Consequently, no single biomarker can accurately characterize iron status in isolation. These limitations underscore the need for integrated diagnostic approaches that simultaneously consider iron metabolism and inflammatory status. Such approaches provide the conceptual basis for the development and refinement of models such as BRINDA.

## 5. Inflammatory Biomarkers

The assessment of inflammatory status relies primarily on acute-phase proteins such as CRP and AGP, which form the basis of the adjustment models proposed by BRINDA [2,4]. CRP is particularly useful for detecting metabolic inflammatory burden, whereas AGP may better capture more sustained inflammatory responses, including those associated with chronic infections and some non-communicable conditions related to obesity and metabolic dysfunction [33,34,35].

However, the ability of these biomarkers to fully capture the complexity of the inflammatory response remains limited. In certain infectious conditions, such as malaria, correction models based on CRP and AGP do not always adequately adjust ferritin concentrations, suggesting the influence of additional factors, including environmental and sociodemographic determinants [36].

Pro-inflammatory cytokines are important mediators of the interplay between inflammation and metabolic homeostasis. IL-6 is closely involved in the regulation of hepcidin and systemic iron homeostasis, thereby influencing circulating iron availability [37]. In parallel, metabolic disorders such as diabetes are characterized by chronic low-grade inflammatory activation involving cytokines such as TNF-α and IL-6 and inflammatory signaling pathways, which may contribute to the persistence of metabolic inflammation [38,39].

More recently, derived hematological indices have emerged as integrative markers of systemic inflammation, including the neutrophil-to-lymphocyte ratio (NLR), the systemic inflammation response index (SIRI), and the systemic immune-inflammation index (SII) [40,41,42]. The NLR, calculated as the ratio of neutrophil count to lymphocyte count, reflects the balance between innate inflammatory activation and adaptive immune regulation. The SIRI, calculated as neutrophil count × monocyte count/lymphocyte count, integrates inflammatory and immune cell populations and has been proposed as a marker of systemic inflammatory burden. The SII, calculated as platelet count × neutrophil count/lymphocyte count, incorporates indices that capture the balance between pro-inflammatory and immunoregulatory processes and may provide a more comprehensive assessment of inflammatory status [42].

In a cohort of 4075 adults from the National Health and Nutrition Examination Survey (NHANES), elevated SII was associated with multiple alterations in iron metabolism and demonstrated a stronger association than CRP with both ID and anemia. These findings suggest that integrative inflammatory indices may provide complementary information on the relationship between systemic inflammation and iron status. However, their incremental diagnostic value beyond established inflammatory markers such as CRP and AGP remains to be prospectively validated [43].

Taken together, these findings indicate that inflammatory biomarkers not only reflect inflammatory status but may also influence the interpretation of iron-related parameters. This interdependence underscores the need to understand the mechanistic links between inflammation and iron metabolism, particularly in the context of chronic low-grade inflammation.

## 6. Inflammation–Iron Interaction

The interaction between chronic low-grade inflammation and iron metabolism emerges as a complex and dynamic process involving both systemic and cellular regulatory mechanisms. Chronic inflammation, whether of metabolic or infectious origin, induces hepcidin expression, a key regulator of iron homeostasis, as it promotes iron sequestration in macrophages and hepatocytes and reduces systemic availability of iron [24,44]. This mechanism is central to the development of functional ID.

Traditionally, inflammation has been considered the primary driver of iron dysregulation. However, emerging evidence suggests that this relationship may be bidirectional (Figure 1). Some studies indicate that the contribution of low-grade inflammation to systemic oxidative stress may be modest, whereas iron status, particularly ferritin, is more strongly associated with oxidative damage, suggesting that iron-mediated mechanisms may operate independently of, or downstream from, inflammatory signaling [45].

Ferritin has emerged as an integrative marker of metabolic and inflammatory alterations with clinical manifestations. In children with hepatic steatosis, ferritin levels have been strongly associated with disease severity and metabolic risk burden, supporting its role as a nonspecific marker of metabolic–hepatic dysfunction [46]. Similarly, in adults, lower serum iron levels have been associated with elevated levels of low-grade inflammatory markers, particularly CRP and TNF-α, suggesting a link between chronic inflammatory activation and altered iron metabolism, although these findings must be interpreted cautiously because of the variability of serum iron and its sensitivity to inflammatory states [47].

Longitudinal evidence further supports the complexity of the interaction between iron metabolism and inflammation. In one longitudinal study, iron deficiency defined by elevated sTfR concentrations was associated with progressive increases in IL-6 and CRP, independent of anemia status over time. These findings suggest that the relationship between iron deficiency and inflammation may be bidirectional, although the underlying mechanisms remain uncertain and require confirmation in further longitudinal and mechanistic studies [48].

From a mechanistic perspective, low-grade inflammation also directly modulates cellular iron regulation. Experimental models have shown that exposure to low concentrations of pro-inflammatory cytokines induces activation of gene networks involved in iron uptake and retention, including Hamp, Steap4, and Lcn2. Notably, this response does not necessarily lead to immediate cellular damage but may exert cytoprotective effects against oxidative stress and ferroptosis, suggesting an early adaptive mechanism under metabolic inflammation [49].

In conditions such as metabolic syndrome, chronic low-grade inflammation is also associated with nutritional alterations, including vitamin D deficiency, and with increased levels of biomarkers such as CRP and ferritin. In this context, hyperferritinemia appears to reflect an inflammatory state rather than true iron overload, further complicating the interpretation of iron biomarkers [50].

Overall, current evidence indicates that the interaction between chronic low-grade inflammation and iron metabolism is not linear but represents a bidirectional system, in which inflammation modulates iron distribution, while iron availability, in turn, influences inflammatory responses.

Chronic inflammation induces hepcidin expression, promoting iron sequestration in macrophages and hepatocytes and reducing systemic iron availability, which may lead to functional iron deficiency. These alterations may contribute to oxidative stress and metabolic dysfunction. Conversely, disrupted iron homeostasis may further amplify inflammatory responses, establishing a bidirectional feedback loop.

## 7. Classical BRINDA Approach

Building on the bidirectional interaction between inflammation and iron metabolism, a key challenge has emerged in clinical and population-based settings: how to accurately interpret iron biomarkers in the presence of inflammation.

The BRINDA project developed a regression correction approach to account for inflammation-related changes in iron biomarkers [2,4]. Unlike earlier methods based on dichotomous CRP (>5 mg/L) and AGP (>1 g/L) cutoffs, the current methodology models the continuous relationship between inflammatory biomarkers and micronutrient indicators. Internal reference values are defined using the lowest decile of CRP and AGP concentrations, after which regression coefficients are applied to adjust biomarker concentrations according to each individual’s inflammatory profile. This continuous correction can reduce misclassification of iron status and has been implemented through publicly available BRINDA macros for ferritin, soluble transferrin receptor, retinol-binding protein, zinc, and other biomarkers [4,51].

In Nepal, the use of BRINDA-adjusted ferritin demonstrated that iron deficiency accounted for approximately 30% of anemia cases in children, highlighting the importance of correcting for inflammation to avoid underestimation [52]. Similarly, in settings where infections such as malaria are common, inflammation can substantially alter the interpretation of ferritin, sTfR, and zinc levels, significantly modifying micronutrient biomarker concentrations [53]. However, BRINDA was developed largely from population surveys in which infection-related inflammation was an important concern, and its performance in settings dominated by chronic low-grade metabolic or physiological inflammation remains less well characterized.

The physiology of populations living at high altitude represents an additional layer of complexity because hypoxia-driven erythropoiesis interacts with inflammation and iron metabolism [54,55]. Recent studies, including work from our group, have demonstrated discrepancies between anemia prevalence based on hemoglobin and iron deficiency in Peruvian children, as well as limitations associated with current WHO altitude-adjusted hemoglobin thresholds [56,57].

Despite the advances made possible by the BRINDA approach, a critical gap remains. In routine practice, ferritin is rarely adjusted for inflammation, particularly in low-resource settings. Moreover, there is limited evidence on the performance of simplified strategies, such as CRP-based adjustments, in pediatric populations and in high-altitude environments. Recent studies have also highlighted the limitations of current diagnostic frameworks and the multifactorial nature of anemia, including the roles of iron excess and inflammation [58,59].

These limitations highlight the need to investigate whether established BRINDA adjustment may be complemented by context-informed interpretative approaches capable of addressing the complexity of chronic low-grade inflammation and its interaction with iron metabolism, as considered in the following section.

## 8. A Context-Informed Framework Complementing BRINDA Adjustment

### 8.1. Conceptual Model

We propose a context-informed conceptual framework that complements, rather than modifies or replaces, established BRINDA adjustment. Classical BRINDA is a quantitative regression-correction methodology that adjusts iron biomarkers for inflammation using established inflammatory markers. In contrast, the proposed framework does not introduce a new correction equation or modify the BRINDA regression procedure. Rather, it provides a structured basis for interpreting iron status after established inflammation-adjustment procedures have been applied, particularly in heterogeneous settings where biological, physiological, and environmental factors may influence the interpretation of iron biomarkers [36]. The proposed components should therefore not be interpreted as having established weights, thresholds, correction coefficients, or combined decision rules.

As illustrated in Figure 2, the framework is organized into three complementary levels. First, inflammatory status is assessed using established acute-phase proteins, particularly CRP and AGP. Composite hematological indices such as SII, NLR, and SIRI may provide complementary information on systemic inflammatory activity; however, they should currently be considered candidate adjunctive or investigational markers rather than validated substitutes for CRP or AGP. Importantly, their incremental diagnostic value for iron-status classification beyond established inflammatory markers has not yet been demonstrated [40,41,42,43,60].

Second, iron status is assessed using established biomarkers and interpretative approaches. Ferritin remains the primary indicator of iron stores and, when appropriate, may be adjusted for inflammation using validated methods such as BRINDA. Additional biomarkers, including sTfR, TBI, and TSAT, may provide complementary information on iron availability and functional iron status [26,27,29,30,51]. Hepcidin may provide additional mechanistic information on inflammation-mediated iron restriction; however, its current application remains limited by assay standardization and availability [25].

Third, the resulting iron-status profile is interpreted in relation to relevant contextual modifiers, including altitude, obesity and metabolic status, environmental exposures, infectious burden, and life-course stage. These factors are not proposed as additional correction variables with predetermined weights; rather, they identify biological and epidemiological contexts in which conventional interpretation of iron biomarkers may require particular caution. Importantly, the consideration of contextual variables alongside CRP and AGP is supported by prior BRINDA-related research.

For example, malaria infection has been evaluated as an additional contextual exposure in the interpretation of inflammation-adjusted ferritin. Recent evidence from school-age children in Malawi showed that malaria positivity and recent fever were associated with alterations in micronutrient biomarkers even when inflammation-adjusted ferritin was considered, suggesting that infection-related contextual factors may remain relevant after conventional assessment of inflammation [61]. This example illustrates that contextual variables can be empirically evaluated for their potential contribution to iron-status interpretation without implying that they should be incorporated into BRINDA adjustment as universal correction factors. These three levels are therefore intended to structure interpretation rather than to define a sequential clinical algorithm; no mathematical rule is currently proposed for combining variables across domains.

Accordingly, the proposed framework should be regarded as a structured interpretative and research framework. No validated thresholds, weighting system, correction equation, or clinical decision rules are currently available for combining all of its components. Prospective validation is therefore required before the framework can be considered for individual clinical decision-making or population surveillance. Such studies should directly compare established approaches based on CRP and AGP with models additionally incorporating SII, NLR, SIRI, or selected contextual modifiers to determine whether these components provide incremental value for iron-status classification.

Figure 2 should therefore not be interpreted as a clinical diagnostic algorithm. Rather, it identifies domains that may warrant consideration when interpreting iron biomarkers and provides a structure for future validation studies. Current evidence is insufficient to assign quantitative weights to contextual modifiers or to establish decision thresholds combining classical inflammatory markers, composite hematological indices, and contextual factors. Future prospective studies should determine whether incorporating selected components improves iron-status classification beyond established BRINDA adjustment.

An additional feature of the framework is its recognition of the potentially bidirectional relationship between inflammation and iron metabolism. Although inflammation is a well-established driver of iron redistribution, emerging evidence suggests that alterations in iron status may themselves influence inflammatory and oxidative pathways [45]. This bidirectional relationship further emphasizes the need for caution when interpreting ferritin in inflammatory and metabolic contexts, because ferritin concentrations may reflect both iron stores and inflammatory activity.

To distinguish the nature and strength of the evidence supporting the different components of the proposed framework, Table 2 summarizes whether current support is derived primarily from mechanistic evidence, epidemiological associations, diagnostic or methodological evidence, or direct validation. This distinction is particularly important because biological plausibility or epidemiological association should not be interpreted as evidence of improved diagnostic classification. Established BRINDA components have direct methodological and validation evidence for inflammation adjustment, whereas several composite inflammatory indices and contextual modifiers are supported primarily by mechanistic or epidemiological evidence and therefore remain investigational within the proposed framework.

### 8.2. Application in Complex Physiological Contexts: Altitude as an Illustrative Setting

Peru, where a substantial proportion of the population lives at high altitude, provides a particularly relevant physiological setting for illustrating the proposed framework. Chronic exposure to hypoxia stimulates erythropoiesis and alters iron requirements, utilization, and distribution [16,54]. These physiological adaptations may coexist with inflammatory and nutritional factors, creating a complex environment in which conventional interpretation of hemoglobin and iron biomarkers becomes particularly challenging.

Studies in high-altitude populations have demonstrated substantial discordance between anemia defined by hemoglobin concentration and biochemical evidence of ID, particularly among children [55,56]. These observations indicate that anemia in such settings cannot necessarily be attributed to ID alone and may reflect the combined influence of hypoxia-related hematological adaptation, iron status, inflammation, and other biological factors. In addition, the application of WHO hemoglobin cutoffs in high-altitude populations has been questioned because altitude adjustment may substantially influence anemia prevalence estimates and, consequently, decisions regarding intervention [16].

Within this physiological context, low-grade inflammation may coexist with hypoxia and influence iron homeostasis through hepcidin-mediated pathways, potentially reducing circulating iron availability and contributing to functional ID. At the same time, hypoxia-driven erythropoiesis alters iron demand. The coexistence of these mechanisms further complicates the interpretation of ferritin and other iron biomarkers when considered in isolation.

The proposed framework therefore uses altitude as an illustrative contextual modifier rather than as a validated correction variable. It provides a structure for examining how inflammatory status, established iron biomarkers, and altitude-related physiology may interact when interpreting iron status. Whether consideration of altitude or other contextual modifiers improves discrimination between absolute ID, inflammation-mediated functional ID, and hypoxia-related hematological adaptations beyond established BRINDA adjustment remains to be determined in prospective studies.

## 9. Inflammation and Childhood Anemia in Peru: Challenges for BRINDA Application

Peru represents a particularly relevant setting for studying the interaction between inflammation and iron metabolism because childhood anemia coexists with heterogeneous environmental and biological conditions, including infectious diseases, obesity, high-altitude hypoxia, and marked ecological diversity.

National surveys have consistently documented a high prevalence of anemia among children aged 6–59 months despite more than a decade of national supplementation and anemia-control programs (Figure 3A). Although anemia prevalence declined between 2010 and 2021, it increased again after the COVID-19 pandemic, reaching nearly 30% in 2024. Throughout the study period, rural populations consistently exhibited substantially higher anemia prevalence than urban populations, reflecting persistent geographic and socioeconomic disparities [62].

Reported consumption of iron supplements in the last 7 days increased progressively over the same period, particularly in rural areas, although a temporary decline occurred during the COVID-19 pandemic because of disruptions in healthcare delivery (Figure 3B). These parallel population-level trends are ecological and descriptive and should not be interpreted as evidence of individual-level effectiveness or causality between reported iron supplement consumption and anemia prevalence.

Nevertheless, improvements in reported consumption of iron supplements in the last 7 days have not been accompanied by proportional reductions in anemia prevalence, a pattern consistent with the possibility that factors beyond iron deficiency contribute to the persistent burden of childhood anemia in Peru [62].

One important limitation of current national surveillance is the lack of routine assessment of iron and inflammatory biomarkers. Large population-based surveys generally include hemoglobin measurements but do not systematically measure ferritin, AGP, CRP, hepcidin, or other biomarkers required for comprehensive assessment of iron status and BRINDA-type adjustments.

Evidence from Peruvian studies supports this complexity, demonstrating considerable heterogeneity in the contribution of iron deficiency and inflammation across different ecological settings [16,55,57,58].

A recent analysis conducted among young children residing in Puno, Peru, estimated that 27.5% of anemia cases were attributable to iron deficiency, whereas inflammation accounted for 45.9% and other causes for the remaining 26.6% [57].

Similarly, studies conducted in high-altitude populations have shown that many children classified as anemic according to hemoglobin do not meet biochemical criteria for iron deficiency, highlighting the limitations of relying exclusively on hemoglobin for anemia diagnosis [16].

These findings also illustrate the challenges of interpreting ferritin in settings where chronic low-grade inflammation may coexist with infections, environmental enteropathy, obesity, and hypoxia-related physiological adaptations. Under these conditions, ferritin concentrations may be elevated independently of iron stores, increasing the risk of misclassifying iron status if inflammatory adjustment is not performed.

Although the BRINDA approach represented a major advance by correcting ferritin concentrations for inflammation, its implementation in Peru remains limited because AGP and other inflammatory biomarkers are not routinely collected in national surveys or clinical practice. Consequently, additional strategies integrating accessible inflammatory markers with contextual variables such as altitude and metabolic status warrant investigation to determine whether they improve interpretation of iron biomarkers in resource-limited settings [40,41,42,43,60].

Future population-based studies incorporating ferritin, inflammatory biomarkers, and contextual variables would allow direct comparison of conventional ferritin interpretation, BRINDA-adjusted estimates, and context-informed interpretative approaches. Peru therefore provides an important illustrative example of the epidemiological, environmental, and physiological complexity of childhood anemia. The Peruvian evidence presented here illustrates the rationale for considering contextual heterogeneity when interpreting iron status but should not be regarded as validation of the proposed framework or as evidence of improved diagnostic performance.

## 10. Clinical and Public Health Implications

The proposed context-informed framework has important clinical and public health implications, particularly in settings characterized by heterogeneous epidemiological, environmental, and physiological conditions. In clinical practice, the interpretation of iron biomarkers in isolation, particularly ferritin, may lead to clinically relevant misclassification.

This is especially relevant in vulnerable populations such as children and pregnant women, in whom ID, infection, inflammation, and increased physiological iron requirements frequently coexist. In these contexts, inflammation-induced elevations in ferritin may mask underlying ID. Conversely, hyperferritinemia may be misinterpreted as iron overload when it reflects inflammation, metabolic dysfunction, or altered iron distribution. Both scenarios may lead to inappropriate decisions, including underdiagnosis of iron deficiency, unnecessary restriction of iron supplementation, or indiscriminate use of iron interventions when anemia is driven by non-iron causes.

By integrating inflammatory status, iron biomarkers, and contextual modifiers within a unified conceptual structure (Figure 2), the proposed context-informed framework identifies domains that may be relevant to iron-status classification in heterogeneous populations. However, whether incorporating these additional domains improves classification beyond established approaches has not yet been demonstrated. Accordingly, patterns such as absolute ID, inflammation-mediated functional ID, and inflammatory states without biochemical evidence of iron deficiency should be regarded as conceptual interpretative phenotypes rather than validated diagnostic categories. The biomarker combinations and threshold values required to distinguish these phenotypes have not been established and require prospective empirical validation.

At the population level, failure to account for inflammation remains an important source of bias in estimating the prevalence of ID. Misclassification may result in suboptimal planning of nutritional interventions and inefficient allocation of resources, particularly in low- and middle-income countries [63,64,65,66]. In addition, hemoglobin-based anemia surveillance alone cannot determine whether anemia is caused by iron deficiency, inflammation, altitude-related physiology, infection, or other etiologies [67].

The Peruvian context provides a particularly illustrative example of this complexity. The coexistence of diverse ecological regions such as coastal, Andean, and Amazonian regions creates distinct epidemiological profiles that may differentially influence iron metabolism. In coastal areas, the increasing prevalence of obesity and metabolic disorders may contribute to chronic low-grade inflammation.

In the Andean highlands, hypoxia-driven erythropoiesis interacts with inflammation and iron metabolism, complicating the interpretation of hemoglobin and iron biomarkers. In contrast, Amazonian populations are more frequently exposed to infectious diseases, where acute and chronic inflammation may predominate. These contrasting scenarios highlight the limitations of uniform diagnostic approaches and underscore the need for context-specific interpretation [63,64,65].

If prospectively validated, selected components of the proposed context-informed framework could be evaluated for their potential utility in nutritional surveillance or clinical assessment. Future studies should determine whether their incorporation improves iron-status classification beyond established approaches and whether any such improvement translates into more appropriately targeted supplementation strategies or other clinically and programmatically meaningful outcomes.

Such implementation would require demonstration of incremental diagnostic value, reproducibility across populations, feasibility, and clinically or programmatically meaningful thresholds. Particular attention should be given to whether accessible biomarkers, including CRP and derived hematological indices, provide additional value beyond established BRINDA adjustment in resource-limited settings.

Overall, the clinical and public health implications of this proposed context-informed framework lie in moving from isolated biomarker interpretation toward integrated assessment of iron status, inflammation, and the local context. Whether such an approach reduces misclassification, improves etiologic assessment of anemia, or leads to more appropriate interventions in heterogeneous populations remains to be established in prospective validation and implementation studies.

## 11. Conclusions

The BRINDA approach represents a major advance in accounting for the effects of inflammation on iron biomarkers. However, iron-status assessment remains challenging in heterogeneous settings where chronic low-grade inflammation, obesity, metabolic dysfunction, recurrent infections, environmental exposures, and physiological modifiers such as high-altitude hypoxia may coexist.

The context-informed framework proposed in this review complements the established BRINDA adjustment by organizing these factors into three domains: assessment of inflammation, interpretation of iron biomarkers using established methods, and consideration of contextual modifiers. The framework is conceptual and hypothesis-generating; it does not provide validated weights, thresholds, correction equations, or clinical decision rules for combining these domains.

Future research should prospectively evaluate whether incorporating selected inflammatory and contextual variables provides incremental diagnostic value beyond established BRINDA adjustment. External validation across populations with different infectious, metabolic, environmental, and altitude-related exposures will be essential before the framework can be considered for clinical decision-making or population surveillance.

## Figures and Tables

**Figure 1:**
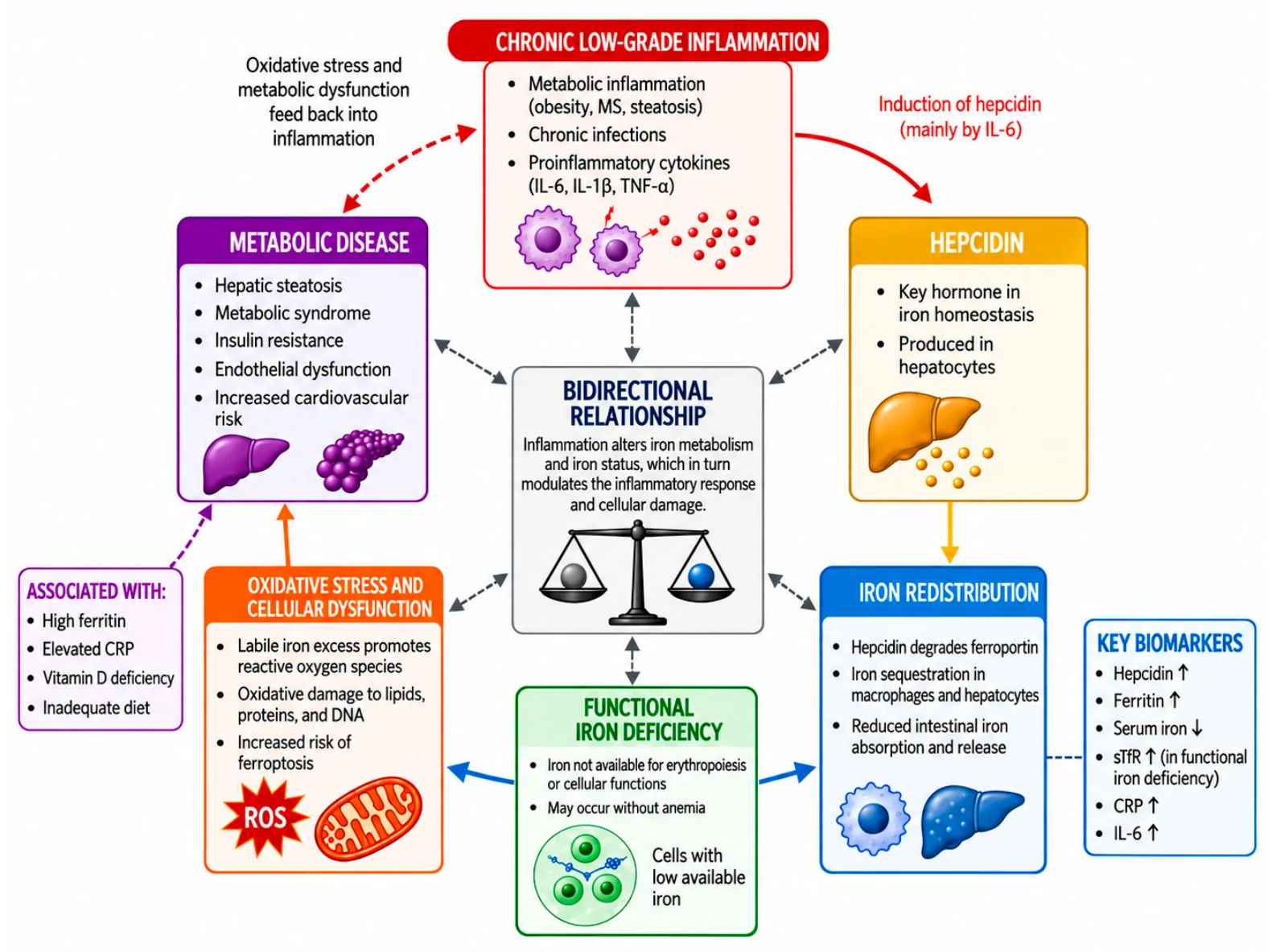
Bidirectional interaction between chronic low-grade inflammation and iron metabolism. MS: metabolic syndrome; IL: interleukin; TNF-α: tumor necrosis factor-alpha; sTfR: soluble transferrin receptor; CRP: *C*-reactive protein; ROS: reactive oxygen species. Solid arrows indicate proposed directional relationships among inflammation, hepcidin induction, iron redistribution, functional iron deficiency, oxidative stress, and metabolic disease. Dashed arrows represent feedback mechanisms and interactions between processes, while double-headed arrows indicate bidirectional relationships. The diagram is conceptual and does not imply established causality.

**Figure 2:**
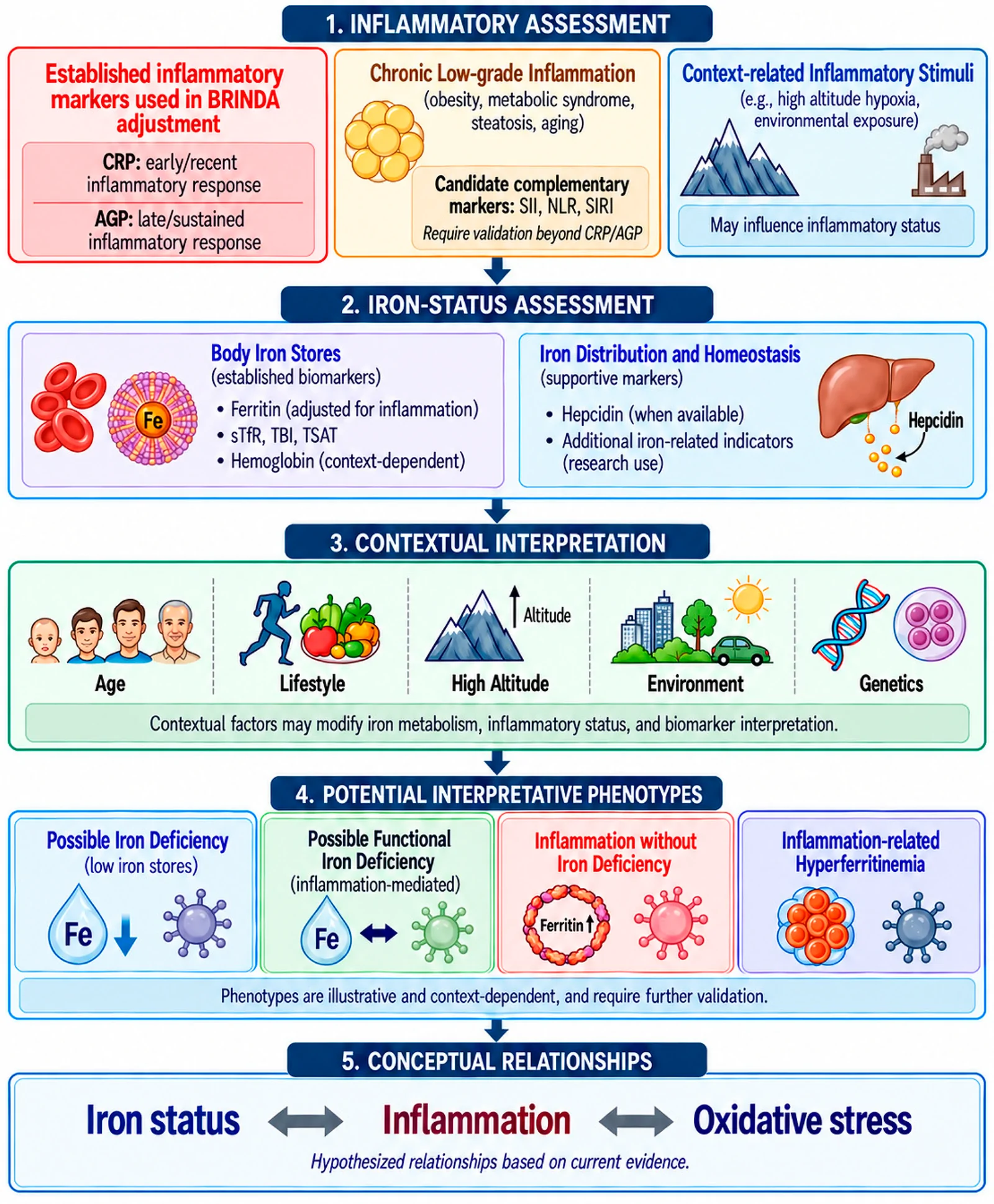
Context-informed conceptual framework complementing established BRINDA adjustment for the interpretation of iron status in heterogeneous settings. The framework integrates three complementary domains: inflammatory assessment, iron-status assessment, and contextual interpretation. Within the inflammatory assessment domain, CRP and AGP are presented as established inflammatory markers used in BRINDA adjustment, reflecting different temporal aspects of inflammation, with CRP more indicative of an early/recent inflammatory response and AGP of a later/sustained response. Chronic low-grade/metabolic inflammation is shown separately, and SII, NLR, and SIRI are presented as candidate adjunctive markers requiring further validation beyond CRP/AGP. Established biomarkers and methods are distinguished conceptually from candidate adjunctive markers and contextual modifiers that require further validation. The framework is intended as an interpretative and research structure rather than a validated diagnostic algorithm. CRP: *C*-reactive protein; AGP: α1-acid glycoprotein; SII: systemic immune-inflammation index; NLR: neutrophil-to-lymphocyte ratio; SIRI: systemic inflammation response index; sTfR: soluble transferrin receptor; TBI: total body iron; TSAT: transferrin saturation. Solid downward arrows indicate the proposed sequence of assessment and interpretation, from inflammatory and iron-status assessment to contextual interpretation and potential phenotypes. Double-headed arrows indicate hypothesized bidirectional relationships among iron status, inflammation, and oxidative stress. These relationships are conceptual and do not imply established causality.

**Figure 3:**
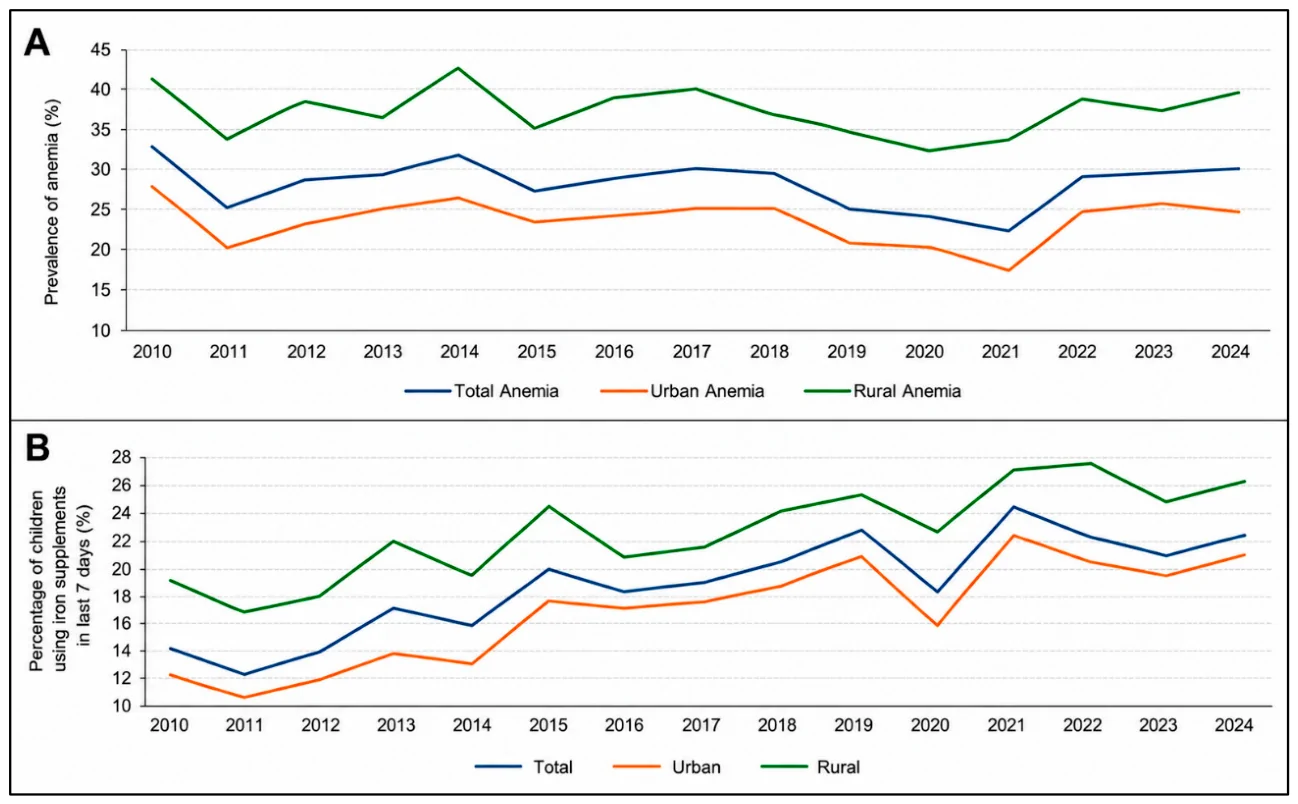
(**A**) Trends in anemia prevalence among children aged 6–59 months in Peru, 2010–2024. (**B**) Trends in the percentage of children aged 6–59 months who reported consuming iron supplements in the last 7 days in Peru, 2010–2024. These ecological trends are presented for population-level context and do not establish individual-level effectiveness or causal relationships. Source: authors’ construction using data from national surveillance reports (INEI-ENDES, 2010–2024) [62].

**Table 1:** Main characteristics and limitations of iron biomarkers under chronic low-grade inflammation.

Biomarker	Reflects	Main Limitation
Ferritin	Body iron stores	Acute-phase reactant; increases during inflammation, reducing specificity for iron deficiency.
sTfR	Cellular iron demand	May be influenced by infection, increased erythropoiesis, and hematological disorders.
TSAT	Circulating iron availability	High biological variability; influenced by inflammation, diurnal variation, and recent iron intake.
TBI	Storage and functional iron compartments	Depends on ferritin and sTfR assumptions that may not hold during inflammation.
Hepcidin	Systemic iron regulation	Assays lack standardization and are not routinely available in clinical practice.

sTfR: soluble transferrin receptor. TSAT: transferrin saturation. TBI: Total Body Iron.

**Table 2:** Type, current evidentiary support, and principal evidence gaps for major components of the context-informed framework.

Component	Primary Evidence Supporting Its Role	Current Interpretation Within the Framework	Key Evidence Gap	Representative Refs.
CRP/AGP	Methodological, epidemiological, validation	Established inflammatory markers used in BRINDA adjustment	Performance may vary across inflammatory contexts	[36,51]
Ferritin	Biological, epidemiological, diagnostic, guideline-based	Established primary indicator of iron stores; requires consideration of inflammation	Reduced specificity for ID during inflammation	[2,18,19,20,21,22,23]
sTfR	Biological, epidemiological, methodological	Complementary marker of cellular iron demand; BRINDA adjustment methods available	May be influenced by infection and erythropoiesis	[26,51]
TSAT/TBI	Physiological, epidemiological, diagnostic/methodological	Complementary indicators of circulating or total-body iron status	Interpretation may be affected by inflammation and underlying biomarker assumptions	[16,27,28,29,30]
Hepcidin	Strong mechanistic; limited diagnostic evidence	Mechanistically relevant to inflammation-mediated iron restriction	Assay standardization, availability, and validated diagnostic thresholds	[24,25,44]
SII	Epidemiological association	Candidate adjunctive inflammatory marker	Incremental diagnostic value beyond CRP/AGP has not been demonstrated	[42,43]
NLR/SIRI	Epidemiological association; general inflammatory evidence	Candidate/investigational inflammatory indices	Limited direct evidence for iron-status classification and no demonstrated incremental value beyond CRP/AGP	[32,40,41,60]
Altitude/hypoxia	Physiological, epidemiological, clinical observational	Contextual modifier relevant to hemoglobin and iron interpretation	No validated weighting or demonstrated incremental value within the proposed framework	[54,55,56,57]
Obesity/metabolic status	Mechanistic and epidemiological	Contextual modifier associated with chronic low-grade inflammation and altered iron biomarkers	No validated integration rule for iron-status classification	[10,11,12,17,31,46,50]
Environmental/infectious exposures	Epidemiological and population-based	Contextual modifiers that may influence inflammation and biomarker interpretation	Heterogeneous exposures; quantitative contribution and integration remain undefined	[36,53,59,62,63,64,65]

AGP: α1-acid glycoprotein; BRINDA: Biomarkers Reflecting Inflammation and Nutritional Determinants of Anemia; CRP: *C*-reactive protein; ID: iron deficiency; NLR: neutrophil-to-lymphocyte ratio; SII: systemic immune-inflammation index; SIRI: systemic inflammation response index; sTfR: soluble transferrin receptor; TBI: total body iron; TSAT: transferrin saturation. Note: Evidence categories indicate the predominant type of support for each component and do not represent a formal grading of evidence certainty. Mechanistic or epidemiological evidence should not be interpreted as demonstrating incremental diagnostic value. Components classified as candidate or contextual require prospective validation before incorporation into diagnostic or surveillance algorithms.

## Data Availability

No new primary data were collected in this study. The data used to construct Figure 3 were obtained from publicly available reports of the Peruvian Demographic and Family Health Survey (INEI-ENDES, 2010–2024), as cited in the manuscript [62]. The remaining information was obtained from the published literature.

## Supplementary Materials

The following supporting information can be downloaded at: https://www.mdpi.com/article/10.3390/nu18193152/s1, Table S1: Full literature search strategy; Table S2: SANRA checklist.

## Abbreviations

The following abbreviations are used in this manuscript:

ID	Iron deficiency
BRINDA	Biomarkers Reflecting Inflammation and Nutritional Determinants of Anemia
CRP	*C*-reactive protein
AGP	α1-acid glycoprotein
sTfR	Soluble transferrin receptor
SII	Systemic immune-inflammation index
SIRI	Systemic inflammation response index
NLR	Neutrophil-to-lymphocyte ratio
IL-6	interleukin-6
TNF-α	tumor necrosis factor-alpha
ESR	erythrocyte sedimentation rate
TBI	Total body iron
NHANES	National Health and Nutrition Examination Survey
