# Supplementary material for: A Context-Informed Framework for Interpreting Iron Status in Heterogeneous Settings: Insights from Peru
Source: Nutrients. 2026 Sep 24;18(19):3152. doi: 10.3390/nu18193152 (PMC13666880; doi:10.3390/nu18193152)
Supplement: Supplementary file 1 [file nutrients-18-03152-s001.zip › nutrients-4572002-supplementary.pdf]

Supplementary Table S1. Full literature search strategy

Database / source	Domain	Full search strategy	Final search date
**PubMed/MEDLINE**	**D1. Biological mechanisms linking inflammation and iron homeostasis**	(("iron deficiency"[Title/Abstract] OR "iron status"[Title/Abstract] OR ferritin[Title/Abstract] OR hepcidin[Title/Abstract] OR "soluble transferrin receptor"[Title/Abstract] OR sTfR[Title/Abstract] OR "transferrin saturation"[Title/Abstract] OR "total body iron"[Title/Abstract]) AND (inflammation[Title/Abstract] OR inflammatory[Title/Abstract] OR "C-reactive protein"[Title/Abstract] OR CRP[Title/Abstract] OR "alpha-1-acid glycoprotein"[Title/Abstract] OR "alpha 1 acid glycoprotein"[Title/Abstract] OR AGP[Title/Abstract] OR "interleukin-6"[Title/Abstract] OR IL-6[Title/Abstract] OR "tumor necrosis factor"[Title/Abstract] OR TNF[Title/Abstract] OR "oxidative stress"[Title/Abstract] OR ferroptosis[Title/Abstract]))	**09/16/2026**
**PubMed/MEDLINE**	**D2. Epidemiological associations between inflammatory and iron biomarkers**	(("iron deficiency"[Title/Abstract] OR anemia[Title/Abstract] OR anaemia[Title/Abstract] OR ferritin[Title/Abstract] OR "serum iron"[Title/Abstract] OR "iron status"[Title/Abstract]) AND ("C-reactive protein"[Title/Abstract] OR CRP[Title/Abstract] OR "alpha-1-acid glycoprotein"[Title/Abstract] OR AGP[Title/Abstract] OR "systemic immune-inflammation index"[Title/Abstract] OR SII[Title/Abstract] OR "neutrophil-to-lymphocyte ratio"[Title/Abstract] OR NLR[Title/Abstract] OR "systemic inflammation response index"[Title/Abstract] OR SIRI[Title/Abstract] OR "interleukin-6"[Title/Abstract] OR "tumor necrosis factor"[Title/Abstract]))	**09/16/2026**
**PubMed/MEDLINE**	**D3. Inflammation adjustment and iron-status classification**	(("iron deficiency"[Title/Abstract] OR anemia[Title/Abstract] OR anaemia[Title/Abstract] OR ferritin[Title/Abstract] OR "soluble transferrin receptor"[Title/Abstract] OR "total body iron"[Title/Abstract] OR hemoglobin[Title/Abstract] OR haemoglobin[Title/Abstract]) AND (BRINDA[Title/Abstract] OR "Biomarkers Reflecting Inflammation and Nutritional Determinants of Anemia"[Title/Abstract] OR "inflammation adjustment"[Title/Abstract] OR "adjusted for inflammation"[Title/Abstract] OR "inflammation-adjusted"[Title/Abstract] OR "regression correction"[Title/Abstract] OR "iron status classification"[Title/Abstract] OR "hemoglobin cutoff"[Title/Abstract] OR "haemoglobin cutoff"[Title/Abstract]))	**09/16/2026**
**PubMed/MEDLINE**	**D4. Contextual modifiers**	(("iron deficiency"[Title/Abstract] OR anemia[Title/Abstract] OR anaemia[Title/Abstract] OR ferritin[Title/Abstract] OR "iron status"[Title/Abstract]) AND (altitude[Title/Abstract] OR "high altitude"[Title/Abstract] OR hypoxia[Title/Abstract] OR obesity[Title/Abstract] OR "metabolic syndrome"[Title/Abstract] OR metabolic[Title/Abstract] OR infection[Title/Abstract] OR infectious[Title/Abstract] OR malaria[Title/Abstract] OR environmental[Title/Abstract] OR "environmental exposure"[Title/Abstract] OR Peru[Title/Abstract] OR Peruvian[Title/Abstract]))	**09/16/2026**
**Scopus**	**D1. Biological mechanisms linking inflammation and iron homeostasis**	TITLE-ABS-KEY(("iron deficiency" OR "iron status" OR ferritin OR hepcidin OR "soluble transferrin receptor" OR sTfR OR "transferrin saturation" OR "total body iron") AND (inflammation OR inflammatory OR "C-reactive protein" OR CRP OR "alpha-1-acid glycoprotein" OR AGP OR "interleukin-6" OR IL-6 OR "tumor necrosis factor" OR TNF OR "oxidative stress" OR ferroptosis))	**09/16/2026**
**Scopus**	**D2. Epidemiological associations between inflammatory and iron biomarkers**	TITLE-ABS-KEY(("iron deficiency" OR anemia OR anaemia OR ferritin OR "serum iron" OR "iron status") AND ("C-reactive protein" OR CRP OR "alpha-1-acid glycoprotein" OR AGP OR "systemic immune-inflammation index" OR SII OR "neutrophil-to-lymphocyte ratio" OR NLR OR "systemic inflammation response index" OR SIRI OR "interleukin-6" OR "tumor necrosis factor"))	**09/16/2026**
**Scopus**	**D3. Inflammation adjustment and**	TITLE-ABS-KEY(("iron deficiency" OR anemia OR anaemia OR ferritin OR "soluble transferrin receptor" OR "total body iron" OR hemoglobin OR haemoglobin) AND (BRINDA OR "Biomarkers Reflecting Inflammation and Nutritional Determinants of Anemia" OR "inflammation adjustment" OR	**09/16/2026**

	**iron-status classification**	"adjusted for inflammation" OR "inflammation-adjusted" OR "regression correction" OR "iron status classification" OR "hemoglobin cutoff" OR "haemoglobin cutoff"))	
**Scopus**	**D4. Contextual modifiers**	TITLE-ABS-KEY(("iron deficiency" OR anemia OR anaemia OR ferritin OR "iron status") AND (altitude OR "high altitude" OR hypoxia OR obesity OR "metabolic syndrome" OR metabolic OR infection OR infectious OR malaria OR environmental OR "environmental exposure" OR Peru OR Peruvian))	**09/16/2026**
**Additional targeted sources**	**Guidelines, national epidemiological data, and supporting evidence**	WHO guidelines and Peruvian national epidemiological/surveillance reports were reviewed as authoritative sources. Reference lists of key reviews, methodological papers, and relevant primary studies were additionally screened to identify publications pertinent to the predefined conceptual domains.	**09/16/2026**

Searches were designed to identify evidence relevant to four predefined conceptual domains: D1, biological mechanisms linking inflammation and iron homeostasis; D2, epidemiological associations between inflammatory and iron biomarkers; D3, methods for inflammation adjustment and iron-status classification; D4, contextual factors potentially modifying these relationships. No lower publication-date or study-design restriction was applied. Publications in English or Spanish were considered during screening. PubMed/MEDLINE and Scopus were used as complementary bibliographic databases. Reference lists of key reviews, methodological papers, and relevant primary studies were additionally screened, and WHO guidelines and Peruvian national epidemiological reports were considered as targeted authoritative sources. Following relevance-based screening and narrative evidence synthesis, 67 references were included in the final review.

Supplementary Table S2. SANRA checklist

SANRA item	Score	Justification in this review
1. Justification of the article's importance for the readership	**2/2**	The Introduction establishes the public health importance of iron deficiency and anemia, explains the diagnostic difficulties created by inflammation, and identifies the need for improved interpretation of iron biomarkers in heterogeneous epidemiological and physiological settings.
2. Statement of concrete aims or formulation of questions	**2/2**	The review explicitly states its objective: to critically examine the interaction between chronic low-grade inflammation and iron metabolism and to propose a context-informed interpretative framework that complements established BRINDA adjustment without replacing or modifying it.
3. Description of the literature search	**2/2**	PubMed/MEDLINE and Scopus were searched using predefined concepts related to iron status, inflammation, BRINDA, composite inflammatory indices, altitude, hypoxia, and contextual factors. Eligibility criteria, predefined conceptual domains, title/abstract screening, and full-text assessment are described. The final search date and complete database-specific strategies are provided in Supplementary Table S1.
4. Referencing	**2/2**	Statements regarding biological mechanisms, epidemiological associations, biomarker interpretation, BRINDA methodology, and contextual modifiers are supported by appropriate primary studies, reviews, methodological papers, and international guidelines.
5. Scientific reasoning	**2/2**	The manuscript distinguishes mechanistic, epidemiological, methodological, and validation evidence and explicitly cautions that biological plausibility or epidemiological association should not be interpreted as evidence of improved diagnostic performance.
6. Appropriate presentation of data	**2/2**	Quantitative and population-level evidence is presented where relevant, while limitations are explicitly acknowledged. The proposed framework is repeatedly described as conceptual and hypothesis-generating, without validated weights, thresholds, correction equations, or clinical decision rules, and the conclusions remain proportional to the available evidence.
**Total SANRA score**	**12/12**	The manuscript fulfills all six SANRA domains after the methodological revisions described above.

SANRA comprises six items scored from 0 to 2, with a maximum total score of 12. The final revised manuscript was independently assessed by two authors. Differences in individual item scores were discussed and resolved by consensus.
